# Glioblastoma extracellular vesicles influence glial cell hyaluronic acid deposition to promote invasiveness

**DOI:** 10.1093/noajnl/vdad067

**Published:** 2023-05-27

**Authors:** Dominik Koessinger, David Novo, Anna Koessinger, America Campos, Jasmine Peters, Louise Dutton, Peggy Paschke, Désirée Zerbst, Madeleine Moore, Louise Mitchell, Matthew Neilson, Katrina Stevenson, Anthony Chalmers, Stephen Tait, Joanna Birch, Jim Norman

**Affiliations:** Cancer Research UK Beatson Institute, Glasgow, UK; School of Cancer Sciences, University of Glasgow, Glasgow, UK; Department of Neurosurgery, Freiburg University Hospital, Freiburg, Germany; Cancer Research UK Beatson Institute, Glasgow, UK; Cancer Research UK Beatson Institute, Glasgow, UK; School of Cancer Sciences, University of Glasgow, Glasgow, UK; Cancer Research UK Beatson Institute, Glasgow, UK; Cancer Research UK Beatson Institute, Glasgow, UK; School of Cancer Sciences, University of Glasgow, Glasgow, UK; Cancer Research UK Beatson Institute, Glasgow, UK; Cancer Research UK Beatson Institute, Glasgow, UK; School of Cancer Sciences, University of Glasgow, Glasgow, UK; Cancer Research UK Beatson Institute, Glasgow, UK; Cancer Research UK Beatson Institute, Glasgow, UK; Cancer Research UK Beatson Institute, Glasgow, UK; School of Cancer Sciences, University of Glasgow, Glasgow, UK; School of Cancer Sciences, University of Glasgow, Glasgow, UK; Cancer Research UK Beatson Institute, Glasgow, UK; School of Cancer Sciences, University of Glasgow, Glasgow, UK; School of Cancer Sciences, University of Glasgow, Glasgow, UK; Cancer Research UK Beatson Institute, Glasgow, UK; School of Cancer Sciences, University of Glasgow, Glasgow, UK

**Keywords:** astrocytes, extracellular matrix, extracellular vesicles, GBM, invasion

## Abstract

**Background:**

Infiltration of glioblastoma (GBM) throughout the brain leads to its inevitable recurrence following standard-of-care treatments, such as surgical resection, chemo-, and radiotherapy. A deeper understanding of the mechanisms invoked by GBM to infiltrate the brain is needed to develop approaches to contain the disease and reduce recurrence. The aim of this study was to discover mechanisms through which extracellular vesicles (EVs) released by GBM influence the brain microenvironment to facilitate infiltration, and to determine how altered extracellular matrix (ECM) deposition by glial cells might contribute to this.

**Methods:**

CRISPR was used to delete genes, previously established to drive carcinoma invasiveness and EV production, from patient-derived primary and GBM cell lines. We purified and characterized EVs released by these cells, assessed their capacity to foster pro-migratory microenvironments in mouse brain slices, and evaluated the contribution made by astrocyte-derived ECM to this. Finally, we determined how CRISPR-mediated deletion of genes, which we had found to control EV-mediated communication between GBM cells and astrocytes, influenced GBM infiltration when orthotopically injected into CD1-nude mice.

**Results:**

GBM cells expressing a p53 mutant (p53^R273H^) with established pro-invasive gain-of-function release EVs containing a sialomucin, podocalyxin (PODXL), which encourages astrocytes to deposit ECM with increased levels of hyaluronic acid (HA). This HA-rich ECM, in turn, promotes migration of GBM cells. Consistently, CRISPR-mediated deletion of *PODXL* opposes infiltration of GBM in vivo.

**Conclusions:**

This work describes several key components of an EV-mediated mechanism though which GBM cells educate astrocytes to support infiltration of the surrounding healthy brain tissue.

**Key Points**

- The p53^R273H^ oncogene encourages GBM cells to release EVs containing podocalyxin.
- Podocalyxin-containing EVs from GBM increase hyaluronic acid production by astrocytes.
- Hyaluronic acid production by astrocytes drives GBM migration.

**Importance of the Study**

The infiltrative behavior of glioblastoma (GBM) leads to widespread dissemination of cancer cells throughout the brain. Thus, even following successful resection of the primary tumor these disseminated cells inevitably contribute to post-surgical relapse. In this study, we have discovered a new mechanism through which GBM can release small extracellular vesicles (EVs) to reprogram extracellular matrix (ECM) production by astrocytes in a way that supports increased invasive behavior of the GBM cells. Moreover, we have discovered several key components of the pathway which contribute to this EV-mediated GBM–glial cell communication. Principal among these, we show that a particular mutant of the p53 tumor suppressor, p53^R273H^ drives the release of EVs which foster the deposition of pro-invasive ECM by astrocytes. This study provides mechanistic insight into why brain tumors expressing p53^R273H^ are associated with particularly poor patient survival and highlights the possibility of deploying agents which target astrocyte ECM deposition to reduce the morbidity of p53^R273H^-expressing GBM.

Glioblastoma (GBM) exhibit invasive/infiltrative behavior which is largely responsible for the intractable nature of the disease.^1^ At diagnosis GBM usually display widespread infiltration of the surrounding brain tissue which precludes in toto resection and focused radiotherapy and, therefore, leads to relapse. Furthermore, widespread insinuation of invasive cells into brain tissue is responsible for many of the neurological and cognitive symptoms which contribute to GBM’s morbidity.^2^ Thus, an understanding of the mechanisms though which GBM acquire infiltrative/invasive characteristics, and the cellular mechanisms involved in this, is necessary to assist development of pharmacological strategies to control the disease following resection both before and following chemo and radiotherapy—thus improving accessibility for treatment in the case of relapse and ameliorating its associated neurological and cognitive dysfunction.

The ways in which cancers influence the microenvironment in distant organs to prime these for metastatic colonization are now becoming clear. Soluble components of the cancer secretome can mobilize myeloid cell populations to metastatic target organs to generate immunosuppressive microenvironments.^3^ Additionally, tumor-derived extracellular vesicles (EVs) can prime metastatic niches via alterations to the vasculature and recruitment of myeloid cells with immunosuppressive phenotypes. Furthermore, tumor EVs can alter the extracellular matrix (ECM) within organs such as the liver and lung to generate pro-invasive microenvironments which favor metastatic seeding.^4,5^

Infiltrative behavior in GBM can be mediated via both cell autonomous and complex intercellular communication processes involving alterations in the nearby and distant brain microenvironment. Invading glioma cells produce a range of protrusion types—including tumor microtubes and tunneling nanotubes—to form functional intercellular communication networks which alter the local and wider brain microenvironment to favor infiltration and therapy resistance.^6,7^ EVs also mediate communication between GBM cells and the brain microenvironment in a way that would be expected to favor tumor cell proliferation, angiogenesis, and immune infiltration.^8,9^ As with metastatic priming, a number of studies now indicate that GBM EVs increase recruitment of myeloid cell populations with tumor promoting and/or immunosuppressive properties.^10–13^ However, despite the importance of the ECM in GBM infiltration, it is currently unclear how GBM EVs might alter the brain ECM, and what the role of the brain’s main ECM-depositing cell type, the astrocyte, might be in this regard. Therefore, we have studied how an oncogene—the p53^R273H^ mutant—which is closely associated with poor clinical outcomes—alters the constitution of EVs released by patient-derived glioma cells. We report that EVs released by p53^R273H^-expressing GBM cells are responsible for enabling their invasive behavior in vivo, and this is due to the ability of these EVs to influence the hyaluronic acid (HA) content of ECM deposited by astrocytes.

## Materials and Methods

### Patient-Derived GBM Cells

The E2 and G7 GBM cell lines were obtained from Colin Watts (Cambridge, UK, now Birmingham, UK) and chosen for their characteristic growth pattern in CD1-nude mice xenografts.^14^ Cells were originally isolated from fresh tumor tissue from anonymized patients diagnosed with GBM and continued expression of GBM-characteristic cellular markers was compared with previous publications using these cell lines.^15–18^ GBM cells were cultured on Matrigel (diluted 1:40) under serum-free conditions in adDMEM/F12, 1% B27, 0.5% N2, 4 µg/mL heparin, 10 ng/mL fibroblast growth factor 2 (bFGF), 20 ng/mL epidermal growth factor (EGF), and 1% l-glutamine. The U373-MG GBM cell line was cultured in DMEM containing 10% fetal calf serum on uncoated plastic surfaces.

### CRISPR and Transfections

For CRISPR/cas9-mediated gene knockouts, the following guide sequences were cloned into the lentiCRISPR vector (Addgene plasmid #52961—deposited by the Zhang lab^19^):

TP53 #1 forward CACCGCGCTATCTGAGCAGCGCTCA
TP53 #1 reverse AAACTGAGCGCTGCTCAGATAGCGC
TP53 #2 forward CACCGCCCCGGACGATATTGAACAA
TP53 #2 reverse AAACTTGTTCAATATCGTCCGGGGC
PODXL #1 forward CACCGCAGCTCGTCCTGAACCTCAC
PODXL #1 reverse AAACGTGAGGTTCAGGACGAGCTGC
PODXL #2 forward CACCGGGTGTTCTCAATGCCGTTGC
PODXL #2 reverse AAACGCAACGGCATTGAGAACACCC
Rab35 #1 forward CACCGCTTGAAATCCACTCCGATCG
Rab35 #1 reverse AAACCGATCGGAGTGGATTTCAAGC
Rab35 #2 forward CACCGGAAGATGCCTACAAATTCGC
Rab35 #2 reverse AAACGCGAATTTGTAGGCATCTTCC

Active lentiviruses were produced using HEK293FT cells as the packing line. E2 GBM cells were plated onto Matrigel, transduced with lentiviruses and selected using puromycin (1 µg/mL) or blasticidin (5 µg/mL).

For overexpression of PODXL, the sequence for human PODXL (hPODXL) was cloned into the pQCXlZ-eGFP-C1 retroviral vector (a gift from David Bryant, Glasgow, UK) and Phoenix-Ampho cells were used as the host packaging line. E2 GBM cells were plated onto Matrigel, transduced with retroviruses and selected using Zeocin (1 mg/mL).

### EV Purification and Nanoparticle Tracking Analysis

EVs were collected via differential centrifugation of cell-conditioned medium as described previously.^5,20^ Nanoparticle tracking analysis was carried out using the NanoSight LM10 instrument (Malvern Panalytical) according to the manufacturer’s instructions. To measure uptake by astrocytes, purified EVs were labeled by incubation with PHK67 (2 µM) for 5 min. Excess dye was removed by ultracentrifugation (100 000*g* for 70 min) and labeled EVs were added to primary cultured astrocytes for 24 hours. Recipient astrocytes were then analyzed using flow cytometry.

### ECM Generation

Primary rat astrocytes were grown to 80% confluence in 15 cm dishes before being incubated in the presence or absence of EVs at a concentration of 1 × 10^9^ particles/mL for 72 hours. EV-treated astrocytes were re-plated at 1 × 10^6^ cells/well into 6-well dishes precoated with 0.2% gelatin, which was subsequently crosslinked with 1% glutaraldehyde for 30 min, quenched in 1 M glycine for 20 min. Astrocytes were allowed to deposit ECM for 6 days in medium supplemented with 50 µg/mL ascorbic acid in the presence or absence of the DGK-inhibitor R59022 (10 µM) (Sigma) or hyaluronidase (Hase) (50 µg/mL; Type I-S, Sigma H3506) where indicated. ECM was then decellularized by incubation with PBS containing 20 mM NH_4_OH and 0.5% Triton X-100.

### Immunofluorescence Detection of HA and Chondroitin Sulfate Proteoglycan in Astrocyte-Deposited ECM

To visualize HA, samples were incubated with biotin-conjugated HA-binding protein (HABP) (Sigma 385911), followed by streptavidin conjugated to AlexaFluor-488 (ThermoFisher S11223), and for chondroitin sulfate proteoglycan, a mouse monoclonal antibody (Sigma C8035) was used followed by an anti-mouse secondary antibody conjugated to AlexaFluor-488. Fluorescence was visualized was by confocal microscopy using an Olympus Fluoview FV1000 microscope. Z-stacks were acquired from the substrate to the upper surface of the cultures at intervals of 0.5 µm.

### GBM Cell Migration

GBM cells were seeded onto decellularized ECMs or coronal slices from DGKα^+/+^ or DGKα^−/−^ 5- to 8-week female C57Bl/6 mice. GFP-expressing cells were visualized using a Nikon A1R microscope with frames being captured every 10 min for 16 hours.

### Orthotopic Xenografts

Animal experiments were performed under the relevant home office licence (project licence PPL P4A277133) and in accordance with ARRIVE guidelines. All experiments had ethical approval from the University of Glasgow under the Animal (Scientific Procedures) Act 1986 and the EU directive 2010. Seven-week-old, female CD1-nude mice (Charles River) were anesthetized using isoflurane and placed in a stereotactic frame. To prevent eye desiccation, Lacrilube eye cream was applied. For analgesia, diluted Veterigesic was injected subcutaneously at a final dose of 20 µg/kg. The skin of the surgical area was disinfected using Hydrex skin disinfectant. The mouse was then covered using sterile drapes. Prior to incision, anesthesia depth was assessed via pedal response. Subsequently, the skin was incised along the sagittal suture and periosteum was removed using sterile cotton swabs. A burr hole craniotomy was performed using an electric hand drill 3 mm rostral and 2 mm lateral of the bregma over the right hemisphere. Subsequently, 1 × 10^5^ GBM cells were injected (0.2 × 10^5^ cells/µl in PBS at a rate of 2 µl/min for 2.5 min) using a Hamilton syringe inserted 3 mm into the brain. Skin was adapted and Vetbond tissue glue applied. Mice were put in prewarmed recovery cages and continuously monitored until mobile. For postoperative analgesia, mice were provided with Rimadyl-containing drinking water for 48 hours postoperatively. Mice were continuously monitored throughout the course of the experiment, and humanely sacrificed either upon display of neurological (such as hemiparesis or paraplegia) or general (hunched posture, reduced mobility, and/or weight loss >20%) symptoms, or timed end-point of 9 weeks following engraftment of GBM cells. Formalin fixed, 4-µm-thick coronal mouse brain sections were stained for Ki67 as previously described.^14^

## Results

### EVs From Infiltrative GBM Cells Foster a Pro-migratory Microenvironment in Brain Slices

**Figure 1.**
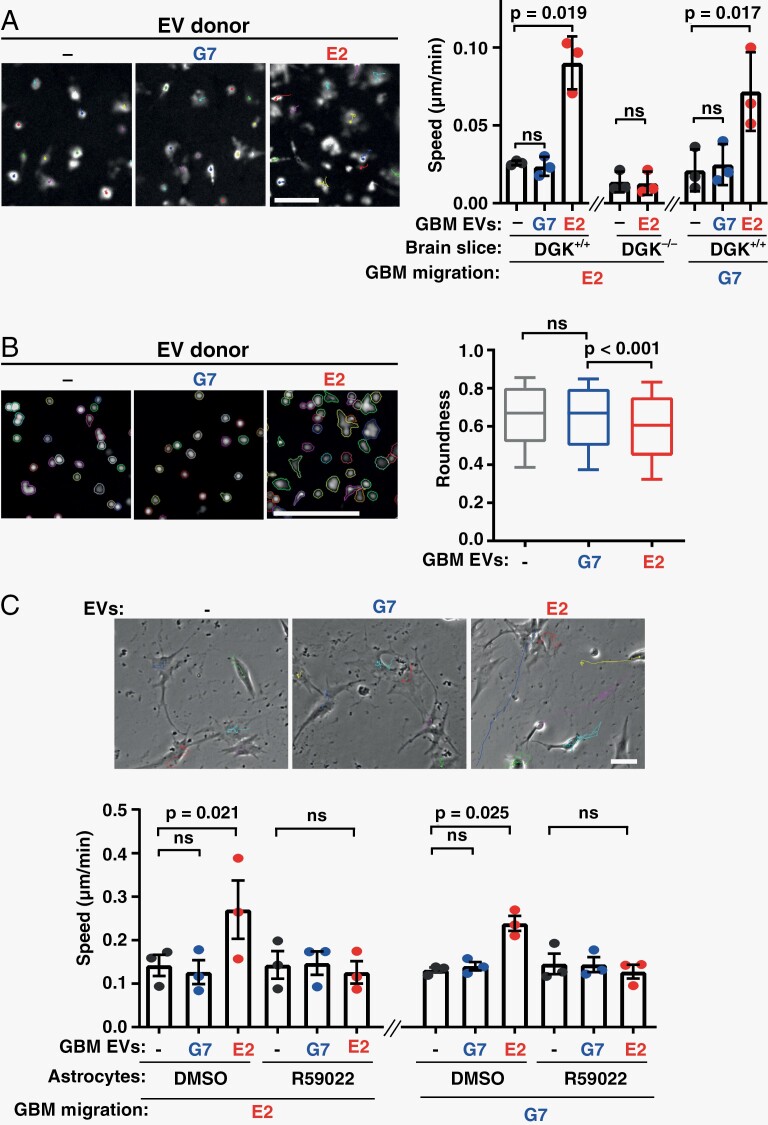
EVs from infiltrative GBM cells foster pro-migratory microenvironments. (A, B) Brain slices from DGK^+/+^ or DGK^−/−^ mice were treated for 72 hours with equal quantities of EVs from E2 or G7 patient-derived glioma stem-like (GBM) cells or left untreated (-). GFP-expressing E2 or G7 cells were then plated onto slices and migration speed (A) and shape (B) determined. In (A) values are mean ± SEM (*n* = 3 independent experiments, paired *t*-test, 60 cell-tracks/condition/experiment). In (B) bars are median, boxes denote interquartile range and whiskers the 10%–90 % range (*n* = 3, unpaired *t*-test). Bars are 50 µm. (C) Astrocytes were treated for 72 hours with EVs from E2 or G7 cells or were left untreated (-). Astrocytes were then allowed to deposit ECM for 6 days in the presence or absence of 10 µM R59022 or vehicle control (DMSO). ECM was decellularized and E2 (left graph and micrographs) or G7 (right graph) cells plated onto these. GBM cell migration was then determined using time-lapse microscopy. Values are mean ± SEM (*n* = 3 independent experiments, paired *t*-test, 60 cell-tracks/condition/experiment). ECM, extracellular matrix; EVs, extracellular vesicles; GBM, glioblastoma.

Two primary patient-derived glioma stem-like cell (GSC) lines, G7 and E2, derived from resected patient tissue^15,18^ were chosen in view of the markedly different invasive/infiltrative characteristics that they display in vivo. Indeed, G7 GSC grow as a solid tumor with moderately invasive margins, whereas E2 disseminate throughout the brain as widely scattered tumor cells.^14^ To determine whether this was owing to their intrinsically different migratory behavior, we plated GFP-expressing G7 and E2 cells onto brain slices from 5- to 8-week Cl57/Bl6 mice and recorded their movement using fluorescence time-lapse microscopy. Surprisingly, both the G7 and E2 cell lines migrated similarly (and poorly) on brain slices (Supplementary Figure S1A) despite their markedly different invasive behavior in vivo.^14^ We hypothesized that the secretome of GBM cells may induce changes in the brain microenvironment which promote invasiveness, and that this may take some time to establish. As EVs are a component of the tumor secretome with key roles in influencing microenvironments both locally and at distance from the primary tumor, we purified EVs from GSC-exposed medium and analyzed these using nanoparticle tracking. G7 and E2 cells released EVs in similar quantities and with indistinguishable size distributions (Supplementary Figure S1B). We then incubated brain slices with EVs from G7 or E2 cells for 72 hours, subsequently plated GFP-expressing E2 or G7 cells onto these and measured their migration. This indicated that pretreatment of brain slices with EVs from E2 (but not G7) cells increased migration of subsequently plated GBM cells (Figure 1A). Consistently, GBM cells plated onto brain slices pretreated with EVs from E2 cells displayed a more invasive phenotype characterized by extension of invasive protrusions which altered the cell shape (Figure 1B).

The lipid kinase, diacylglycerol kinase-α (DGKα) must be expressed in fibroblasts for them to generate pro-invasive microenvironments in response to EVs from primary tumors.^5^ We deployed DGKα knockout (DGKα^−/−^) mice to determine whether this kinase is required for a “recipient” brain cell population to generate a pro-migratory microenvironment in response to GBM EVs. Brain slices from 5- to 8-week DGKα^−/−^ Cl57/Bl6 mice were treated with EVs from E2 cells for 72 hours, and GFP-expressing E2 cells subsequently plated onto these. GBM cells migrated poorly on brain slices from DGKα^−/−^ mice (as they did on slices from DGK^+/+^ mice) and this was unaffected by pretreatment with EVs from E2 cells, indicating that expression of DGKα is required for a brain cell population to generate a pro-migratory microenvironment in response to GBM EVs (Figure 1A).

### EVs From Infiltrative GBM Cells Encourage Astrocytes to Deposit Pro-migratory ECM

A principal task of astrocytes is to deposit ECM in the brain and thus maintain its appropriate microenvironment. Experiments in which we labeled EVs with fluorescent dyes and incubated them with primary cultured astrocytes indicated that these glial cells are capable of assimilating and, therefore, potentially responding to, EVs from both G7 and E2 GBM cells (Supplementary Figure S2). We, therefore, incubated primary cultured astrocytes with EVs from E2 or G7 cells for 72 hours, re-plated the astrocytes and allowed them to deposit ECM for 6 days. Astrocyte cultures were then decellularized and migration of GBM cells on these ECMs was determined using time-lapse microscopy. Pretreatment of astrocytes with EVs from E2 (but not G7) cells prior to ECM generation significantly increased the speed of both E2 and G7 GBM cells plated on these matrices (Figure 1C). Moreover, addition of a DGKα inhibitor (R59022)^21,22^ to the astrocyte cultures opposed the ability of EVs from E2 cells to encourage astrocytes to deposit ECM with increased capacity to support GBM cell migration.

### Deletion of p53^273H^ Reduces the Ability of EVs From GBM Cells to Foster a Pro-migratory Microenvironment

**Figure 2.**
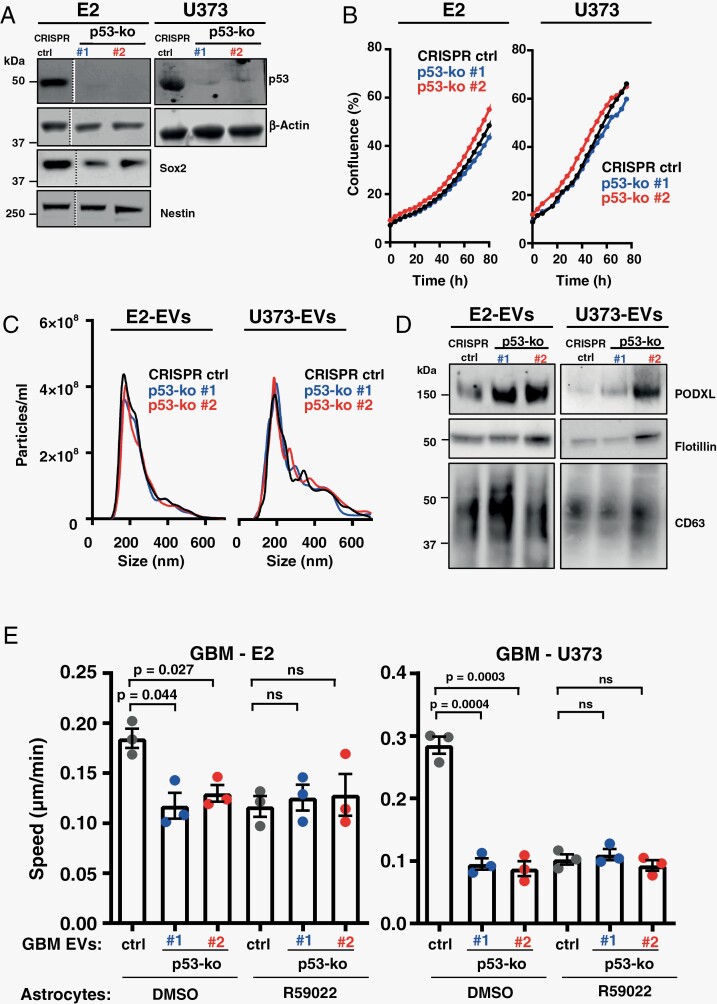
Deletion of p53^R273H^ abrogates the ability of GBM cells to produce EVs which encourage astrocytes to deposit pro-migratory ECM. Characterization of control (CRISPR-ctrl) and p53 knockout (p53-ko #1 and #2) E2 and U373-MG cells: (A) confirms deletion of p53^R273H^ in E2 and U373-MG cells, and expression of stem cell markers, Nestin and SOX2 in E2 cells; (B) indicates that deletion of mutant p53 does not influence cell growth in E2 or U373-MG cells. (C, D) Characterization of EV release by control and p53 knockout E2 and U373-MG cells: The number and size distribution of EVs from CRISPR-ctrl and p53-ko cells were analyzed using nanoparticle tracking (C), and their PODXL, flotillin, and CD63 content was determined by western blot (D). (E) Astrocytes were treated for 72 hours with EVs from control and p53 knockout E2 or U373-MG cells. Astrocytes were then allowed to deposit ECM for 6 days in the presence or absence of R59022, and migration of E2 cells on these ECMs determined as for Figure 1C. Values are mean ± SEM (*n* = 3 independent experiments, paired *t*-test, 60 cell-tracks/condition/experiment). ECM, extracellular matrix; EVs, extracellular vesicles; GBM, glioblastoma.

An analysis of deep-sequencing data revealed that the highly infiltrative E2 cells express the p53^R273H^ mutant (83% of reads), which has well-characterized pro-invasive/metastatic gain-of-function properties^23–26^ (Supplementary table S1A). Contrastingly, expression of mutated p53s in the less invasive G7 cells is less consistent, with reads being detected for various mutations (p.R248Q p.R282W p72r, each approx. 50% of reads) (Supplementary table S1B) for which a connection to invasion and metastasis is not established.^27,28^ To determine whether expression of p53^273H^ contributes to the generation of pro-invasive/migratory microenvironments, we generated clones of E2 cells in which the gene for p53 was targeted using 2 independent CRISPR guide sequences. Western blotting confirmed deletion of p53^R273H^ and that expression of neuronal stem cell markers, Nestin and SOX2, had not been compromised during this procedure (Figure 2A). Moreover, deletion of mutant p53 did not alter proliferation (Figure 2B), nor the quantity or size distribution of EVs released from E2 cells (Figure 2C). As previous work indicates that mutant p53 generates pro-invasive/migratory niches by modulating the quantity of the sialomucin, podocalyxin (PODXL) sorted into EVs,^5^ we determined the influence of p53^273H^ on PODXL levels in EVs from E2 cells. PODXL levels in EVs were increased by deletion of mutant p53^R273H^ (Figure 2D); an observation consistent with previous findings in carcinoma cells, and which moots the probability that EVs from p53-KO E2 cells might have altered ability to foster pro-invasive microenvironments.^5^ Indeed, EVs from p53-KO E2 cells displayed reduced capacity to influence astrocyte ECM deposition in a way that supported migration of GBM cells (Figure 2E). To confirm p53^R273H^’s role in influencing the ability of EVs to foster deposition of pro-migratory ECM, we deployed another GBM cell line, U373-MG—which bears this mutation^29^—and generated clones in which the mutant p53 was targeted using 2 independent CRISPR guides (Figure 2A). Deletion of p53^R273H^ did not alter U373-MG cell proliferation, nor the quantity of EVs that they release (Figure 2B and C). Nevertheless, in a similar manner to that observed for E2 cells, deletion of p53^R273H^ increased the PODXL content of EVs released by U373-MG cells (Figure 2D) and, consistently, reduced their capacity to persuade astrocytes to deposit pro-migratory ECM (Figure 2E). Furthermore, addition of a DGKα inhibitor (R59022) to the astrocyte cultures opposed the ability of EVs from both control U373-MG and E2 cells to evoke deposition of pro-migratory ECM by astrocytes. Finally, treatment of astrocytes with R59022 did not further reduce the (already compromised) migration-supporting properties of ECM deposited by astrocytes treated with EVs from p52^R273H^ CRISPR E2 or U373-MG cells (Figure 2E).

### PODXL Content of GBM EVs Is Critical to Influencing Astrocyte ECM Deposition

**Figure 3.**
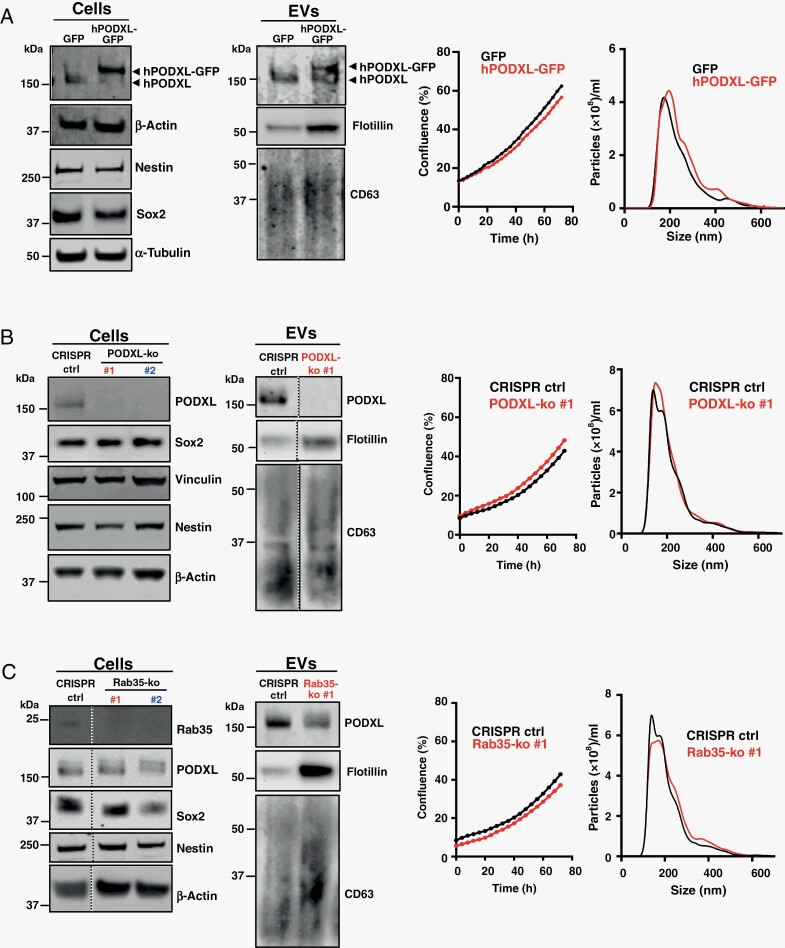
Manipulation of PODXL content in GBM EVs. E2 GBM cells were stably transfected with lentiviral vectors encoding GFP-tagged PODXL (hPODXL-GFP) or GFP (A), or with CRISPR/Cas9 vectors containing 2 independent guide sequences targeting PODXL (PODXL-ko #1 and #2) (B), Rab35 (Rab35-ko#1 and #2) (C), or empty vector control (CRISPR-ctrl). PODXL overexpression, deletion of PODXL and Rab35, expression of stem cell markers, Nestin and SOX2 were confirmed by western blotting. PODXL, flotillin, and CD63 content of EVs was also determined by western blotting. Cell proliferation and the number and size distribution of EV were determined as for Figure 2B and C. EVs, extracellular vesicles; GBM, glioblastoma.

**Figure 4.**
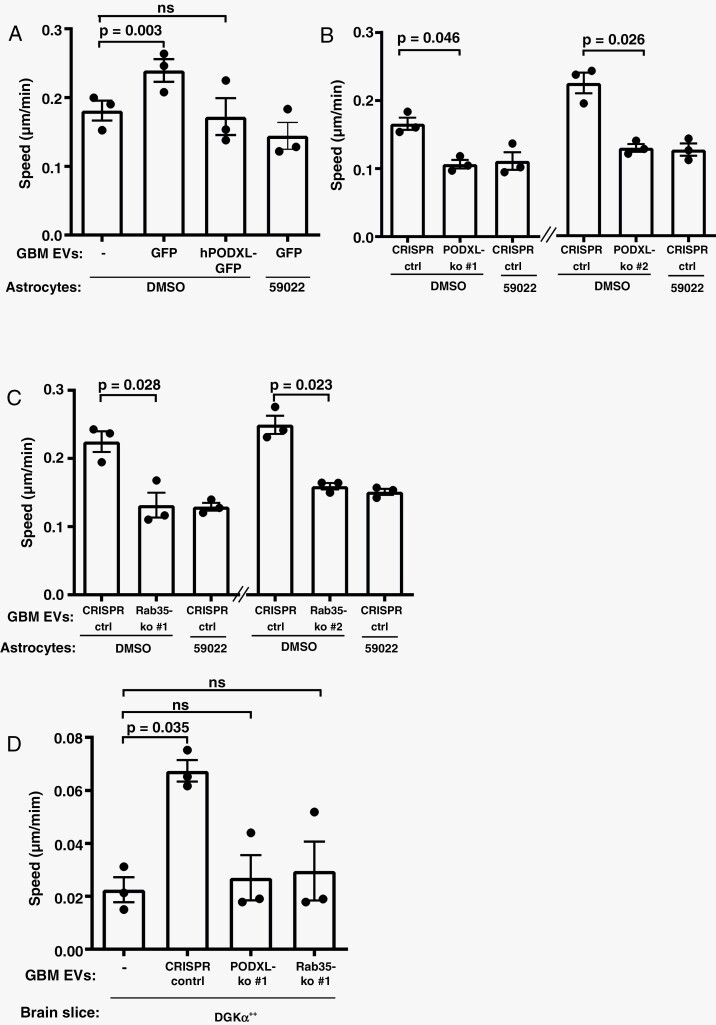
The level of PODXL in GBM EVs is critical for production of pro-migratory ECM by astrocytes. Astrocytes (A–C) or brain slices (D) were treated with EVs from GBM cells in which PODXL or Rab35 levels were manipulated (see Figure 3). Astrocytes were then allowed to deposit ECM for 6 days in the presence or absence of R59022 or vehicle control (DMSO), and migration of E2 cells on these ECMs determined as for Figure 1C. Values are mean ± SEM (*n* = 3 independent experiments, paired *t*-test, 60 cell-tracks/condition/experiment). ECM, extracellular matrix; EVs, extracellular vesicles; GBM, glioblastoma.

The PODXL content of carcinoma cell EVs must be within a certain range for them to encourage fibroblasts to deposit pro-migratory ECM, and expression of mutant p53 functions to move EV PODXL levels into this range.^5^ To determine whether PODXL levels in EVs from GBM is causally linked to their ability to alter ECM deposition by astrocytes, we generated E2 cells in which PODXL levels were either increased (by overexpression) or reduced (using CRISPR). Furthermore, because Rab35 controls PODXL trafficking to EVs,^5^ we also generated Rab35 knockout GBM cells. Immunoblotting confirmed that EV PODXL levels were, respectively, increased or decreased by PODXL overexpression or knockout (Figure 3A and B) and deletion of Rab35 reduced EV PODXL levels (Figure 3C). These manipulations of PODXL and/or Rab35 levels did not alter GBM cell proliferation nor expression of neuronal stem cell markers (Figure 3A–C). We then pretreated astrocytes with EVs from PODXL overexpressing or -deficient, and Rab35 knockout E2 cells for 72 hours, and allowed them to deposit ECM for a further 5 days. Astrocyte-derived ECM was decellularised, and migration of GBM cells on these ECMs determined using time-lapse microscopy. EVs from PODXL overexpressing (Figure 4A) and from PODXL (Figure 4B) or Rab35 (Figure 4C) knockout E2 cells were unable to influence astrocyte ECM deposition in a way that supported increased migration of GBM cells. Finally, we pretreated mouse brain slices with EVs from control and PODXL or Rab35 knockout E2 cells and subsequently plated GBM cells onto these. This indicated that knockout of PODXL or Rab35 opposed the ability of EVs from GBM cells to foster pro-migratory microenvironments in the brain (Figure 4D). These data indicate that expression of p53^R273H^ in GBM cells fosters a pro-invasive brain microenvironment, and that this is via regulation of EV PODXL content and the influence of this on the ECM-depositing capacity of astrocytes.

### EVs Promote GBM Cell Migration by Influencing the HA Content of Astrocyte ECM

**Figure 5.**
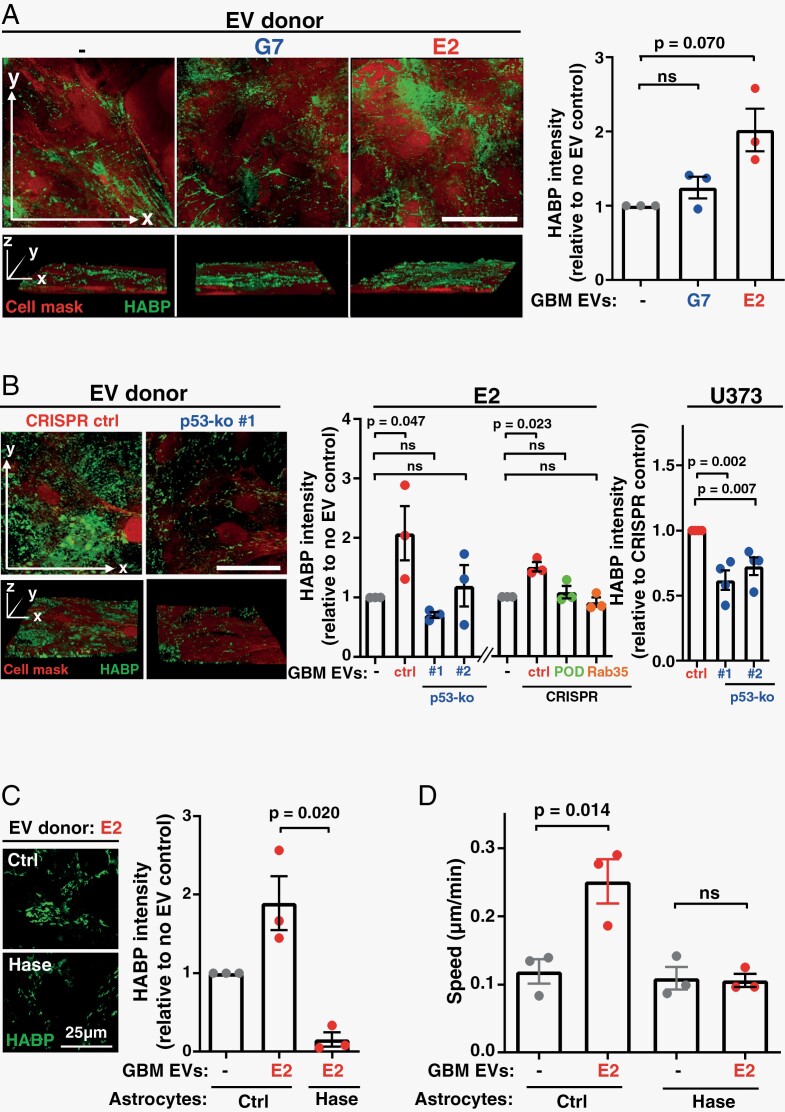
EVs from GBM cells promote GBM migration by influencing HA content of astrocyte ECM. (A, B) Astrocytes were incubated with EVs from G7 or E2 GBM cells (A), and E2 or U373-MG cells (U373) in which p53, PODXL, or Rab35 had been deleted using CRISPR/Cas9 as indicated (B). Astrocytes were then allowed to deposit ECM for 6 days, stained with HABP and cell mask and imaged using fluorescence confocal microscopy; *x*/*y* (upper panels) and *x*/*y*/*z* (lower panels) projections are displayed. Bars 50 µm. HABP was quantified using ImageJ. Values are mean ± SEM (*n* = 3 independent experiments, paired *t*-test; 7 fields/condition/experiment. (C, D) Astrocytes were incubated with EVs from E2 GBM cells and then allowed to deposit ECM for 6 days in the presence or absence of Type I-S Hase (50 µg/mL). HA content of the ECM was assessed as for (A, C), and migration of E2 cells on Ctrl and Hase-treated ECM was determined as for Figure 1C (D). Values are mean ± SEM (*n* = 3 independent experiments, paired *t*-test, 60 cell-tracks/condition/experiment). ECM, extracellular matrix; EVs, extracellular vesicles; GBM, glioblastoma; HA, hyaluronic acid; HABP, hyaluronic acid-binding protein.

EVs from mutant p53-expressing carcinoma cells generate pro-invasive/migratory niches in metastatic target organs (such as the lung), by influencing deposition of fibrillar ECM components, such as fibronectin and collagen.^5^ However, brain ECM is composed primarily of proteo- and glycosamino-glycans and is largely devoid of fibrillar proteins.^30^ We, therefore, used a panel of lectins and reagents recognizing carbohydrate moieties (such as HABP and anti-chondroitin sulfate) to screen for EV-driven alterations to glycan/polysaccharide species in ECM deposited by astrocytes. Most carbohydrate species were not detectably different between the ECM deposited by naive and EV-educated astrocytes (not shown). However, pretreatment of astrocytes with EVs from E2 cells drove a significant increase in the HA (detected by HABP) (Figure 5A), and a moderate, but not significant, decrease the chondroitin sulfate (Supplementary Figure S3A) content of the ECM they deposited. Conversely, pretreatment of astrocytes with EVs from the less invasive G7 GBM cells, or EVs from E2 cells in which either mutant p53^R273H^, PODXL, or Rab35 had been deleted using CRISPR, were ineffective in this regard (Figure 5B). Furthermore, CRISPR-mediated deletion of p53^R273H^ in U373-MG cells significantly reduced the HA content of ECM deposited by astrocytes treated with EVs from this GBM cell line (Figure 5B). HA has a well-established role in promoting migratory and invasive behavior of many cell types^31^—including tumor cells—and examination of Z-projections of 3D confocal image stacks indicated that EVs from E2 cells most significantly increased the HA content of the ECM on upper face of the astrocyte cultures where it is ideally placed to interact with GBM cells plated onto it (Supplementary Figure S3B). We, therefore, allowed astrocytes to deposit ECM in the presence of Hyaluronidase (Hase), which catalyzes hydrolysis of HA, and this led to the deposition of ECM with reduced HA content (Figure 5C). We next plated GBM cells onto ECM generated in the presence and absence of Hase. This indicated that the ability of EV-pretreated astrocytes to generate ECM which supports GBM cell migration was completely ablated by Hase treatment, whilst Hase did not alter the (limited) ability of ECM deposited by EV-naive astrocytes to support GBM cell migration (Figure 5D).

These data indicate that GBM cells expressing the p53^R273H^-mutant release EVs which, by virtue of their PODXL content, encourage astrocytes to deposit ECM with increased HA content which, in turn, promotes GBM cell migration.

### PODXL Drives Mutant p53^R273H^-Driven Infiltrative Behavior of GBM In Vivo

**Figure 6.**
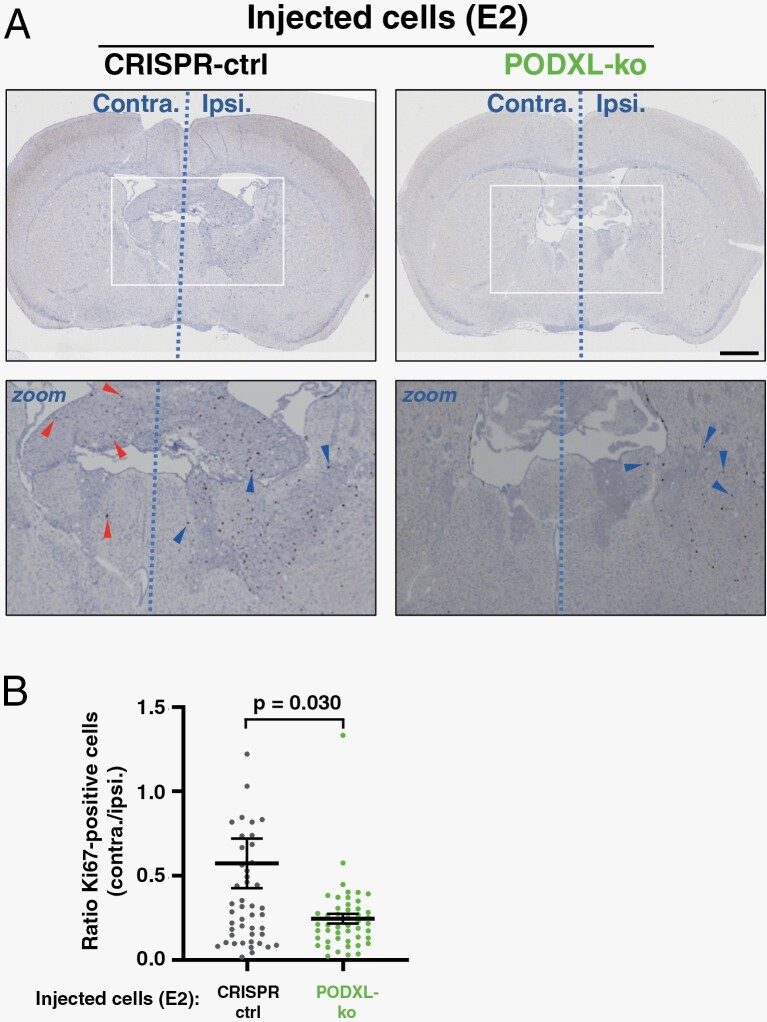
PODXL expression is required for long-range infiltration of GBM cells. CRISPR control (CRISPR-ctrl) or PODXL knockout (PODXL-ko) GBM cells were injected into the right (ipsilateral) striatum of CD1-nude mice. Nine weeks following injection, brains were fixed and the distribution of tumor cells between ipsilateral (Ipsi.) and contralateral (Contra.) determined using Ki67 staining followed by automated image analysis. Cells remaining in the ipsilateral hemisphere and those that have migrated to the contralateral hemisphere are highlighted with blue and red arrows, respectively (A). Ki67-positive cells in the contralateral hemisphere is expressed as a ratio of those in the ipsilateral hemisphere (B). Bars are mean ± SEM. *n* = 10 mice per condition. Statistic is unpaired *t*-test, Welch’s correction. Bar, 1 mm. GBM, glioblastoma.

Having established 3 key components—mutant p53^R273H^, PODXL, and Rab35—necessary for release of EVs which control astrocyte ECM deposition, and described mechanistic details of how they achieve this, we wished to determine whether these components influence GBM infiltration and invasiveness in vivo. We injected mutant p53, PODXL, or Rab35 knockout E2 cells (or CRISPR control) into the right striatum of CD1-nude mice. Nine weeks after injection the number of (proliferating) tumor cells in coronal brain sections (2 consecutive, 50 µm sections per mouse) was quantified by Ki67-staining followed by automated cell counting. We have found that Ki67 staining clearly identifies the nuclei of tumor cells in a way that enables automated analysis. However, Ki67 staining may, due to its inability to detect non-proliferating cells, underestimate the number of invading tumor cells. We, therefore, confirmed that results from Ki67 and human-specific Leukocyte Antigen (to identify the human glioma cells) staining correlated closely.^16^ Moreover, automated counting was undertaken using parameters which excluded Ki67-positive resident brain cells in the subventricular zone. Knockout of mutant p53 or Rab35 reduced tumor growth to an extent which precluded us from determining how these potentially pro-invasive factors might influence infiltration (Supplementary Figure S4). However, as the total number of Ki67-positive GBM cells in the brain was not significantly altered by PODXL knockout (as determined by quantification of either 2 or 5 consecutive 50 µm sections per mouse) (Supplementary Figure S4), we were able to determine how PODXL expression influenced the proportion of GBM cells that had migrated from the ipsilateral to the contralateral (left) hemisphere. This revealed that loss of PODXL significantly reduced the proportion of Ki67-positive GBM cells which moved from the right to the left side of the brain, indicating that PODXL expression contributes to efficient long-range infiltrative/migration of GBM cells (Figure 6A and B).

## Discussion

Communication between cancer cells in the primary tumor and ECM-depositing fibroblasts in metastatic niches is central to metastasis of carcinoma^4,5^ and, in this study, we demonstrate that a process analogous to carcinoma metastatic niche priming may occur as GBM infiltrate the brain (Supplementary Figure S5). Indeed, patient-derived glioma cells can display markedly different migratory behavior within the brain microenvironment, but this owes less to their intrinsic migratory capacity than it does to their influence on ECM deposition by glial cells. Principal among the similarities between this mechanism of glioma infiltration and metastatic niche priming by carcinomas is reliance on an EV-transduced signal emanating from the primary tumor cell which influences the behavior of ECM-depositing cells. This signal is switched-on in both carcinoma and GBM cells by acquisition of a particular p53 mutation—p53^R273H^—which acts in combination with Rab35 to tune the PODXL content of tumor cell EVs into a range that influences ECM deposition by recipient cells—be they astrocytes or fibroblasts. Given the novel gain-of-function that we here describe for p53^R273H^, it is interesting to consider the impact of this particular p53 mutant on glioma progression. This is especially important in light of the invasive nature of both high- and low-grade glioma which prevents curative resection. Indeed, while supratotal resection techniques significantly improve progression-free survival in low-grade glioma, to date no curative treatment is available.^32^ However, any extension of time to recurrence potentially provides a therapeutic window, where inhibiting further invasion of the brain by modulating the microenvironment might facilitate future treatment such as second surgery or irradiation, thus potentially enhancing patient survival. Therefore, given our evidence that p53^R273H^ can engender a pro-invasive brain microenvironment, it was important to determine whether expression of p53^R273H^ might be associated with poor clinical outcomes. Examination of the merged Cell 2016 LGG and GBM dataset using cBioportal indicated that 40% tumors (319/794) displayed mutations of the p53 gene, with the R273H locus being by far the most common p53 mutation—accounting for 22% (69/319) of all p53 mutations (Supplementary Figure S6A). However, the different prognosis of the glioma subtype represented within this dataset prevented us from drawing general conclusions regarding survival. As the database also lacks important information dictating the prognosis of GBM such as tumor location, resection status and adjuvant treatment, we turned to the TCGA LGG PanCancer Atlas dataset^33^ to determine whether IDH-mutant 1p/19q non-codeleted astrocytoma harboring p53^R273H^ might progress more quickly than those bearing other p53 mutations. This indicated that the presence of a p53^273H^ mutation significantly reduced overall median survival by comparison with tumors bearing other p53 mutations (Supplementary Figure S6B). Thus, we submit that the ability of p53^R273H^ to influence the PODXL content of EVs and, thereby, engender a pro-invasive brain microenvironment, may contribute to the particularly fast progression and poor prognosis of glioma bearing this p53 mutant.

Fibroblasts respond to PODXL-containing EVs by upregulating DGKα-dependent integrin recycling; DGKα operates by generating phosphatidic acid in the plasma membrane to enable docking and fusion of endocytic recycling vesicles.^22^ Increased DGKα-dependent integrin recycling then changes alignment of fibrillar ECM deposited by fibroblasts to support tumor cell migration.^5^ Unlike fibroblasts, glial cells do not deposit fibrillar ECM so it is important to consider how DGKα-mediated vesicle trafficking might influence HA deposition by astrocytes. HA synthases are transmembrane proteins which must be plasma membrane localized to coordinate the export and growth of HA chains on the cell surface.^34^ HA synthases, like integrins, cycle between endosomes and the plasma membrane,^35^ and it will be interesting to determine whether EVs from p53^R273H^-expressing glioma influence recycling of these enzymes. Secondly, ECM-associated HA is turned-over by internalization via endocytic (clathrin and non-clathrin dependent) and/or macropinocytic routes followed by Hase-mediated degradation in lysosomes.^34^ In addition to receptor recycling, DKGα also regulates micropinocytosis and endocytosis,^22,36,37^ and it will be interesting to determine whether EVs from glioma influence HA deposition by controlling these internalization mechanisms. Increased HA production has been linked to GBM aggressiveness, via enhanced cancer cell proliferation, chemo- and radio-resistance and invasiveness.^38,39^ This has been attributed to engagement of HA receptors, such as CD44, on tumor cells, and it is likely that the migratory response of GBM cells to HA-rich, astrocyte-deposited ECM is mediated via CD44. HA’s anti-adhesive properties^40^ may also contribute to increased cell migration.

Mobilization of neutrophils and macrophages is key to carcinoma dissemination and metastasis and this, in part, is owing to the immunosuppressive microenvironments that these cells engender. Immunosuppressive myeloid populations, particularly tumor-associated macrophages, also drive glioma progression and therapy resistance. Indeed, interaction of GBM with surrounding astrocytes to induce an immunosuppressive environment has been linked to tumor progression.^41,42^ Interestingly, HA can reeducate tumor-associated macrophages to a tumor-supporting phenotype,^43,44^ and this may promote GBM infiltration. Consistently, studies in which GBM and peripheral blood cells were co-cultured indicated that GBM cells can surround themselves with a halo of HA-rich glycosaminoglycans.^45^ Therefore, increased HA expression by surrounding astrocytes might serve as an additional barrier to invading immune cells while simultaneously promoting GBM invasion.

It is possible that HA may influence glioma infiltration and therapy resistance by promoting tumor cell stemness. HA in the nervous system has its peak during brain development with a subsequent decline to adulthood.^46^ In adult mice, HA is only abundant in the subventricular zone and rostral migratory stream, which are thought to constitute neural stem cell niches.^47^ Therefore, by increasing astrocyte HA secretion, mutant p53-expressing GBM may expand the number of stem cell niches in the adult brain, thus supporting a less proliferative stem-like phenotype capable of evading current standard treatments such as radiotherapy and chemotherapy while simultaneously increasing their infiltration to evade surgical resection.

In conclusion, this study offers a mechanistic explanation for the particularly poor prognosis of IDH-mutant astrocytoma which express the p53^R273H^ mutant and highlights potential druggable targets both within the tumor (Rab35, PODXL) and astrocytes (DGKα) which influence the ECM microenvironment (Supplementary Figure S5). Moreover, our finding that increased HA deposition by astrocytes is key to glioma cell migration will prompt further investigation into the impact of this ECM component on the immune microenvironment in the brain and the regulation of tumor stem cell maintenance and resistance to therapy.

## Supplementary Material

vdad067_suppl_Supplementary_Material
