# Supplementary material for: Glioblastoma extracellular vesicles influence glial cell hyaluronic acid deposition to promote invasiveness
Source: Neurooncol Adv. 2023 May 27;5(1):vdad067. doi: 10.1093/noajnl/vdad067 (PMC10276538; doi:10.1093/noajnl/vdad067)
Supplement: vdad067_suppl_Supplementary_Material [file vdad067_suppl_supplementary_material.docx]

**Supplementary table S1: p53 mutations identified by RNAseq in the E2 and G7 patient-derived glioma stem-like cell lines**

**
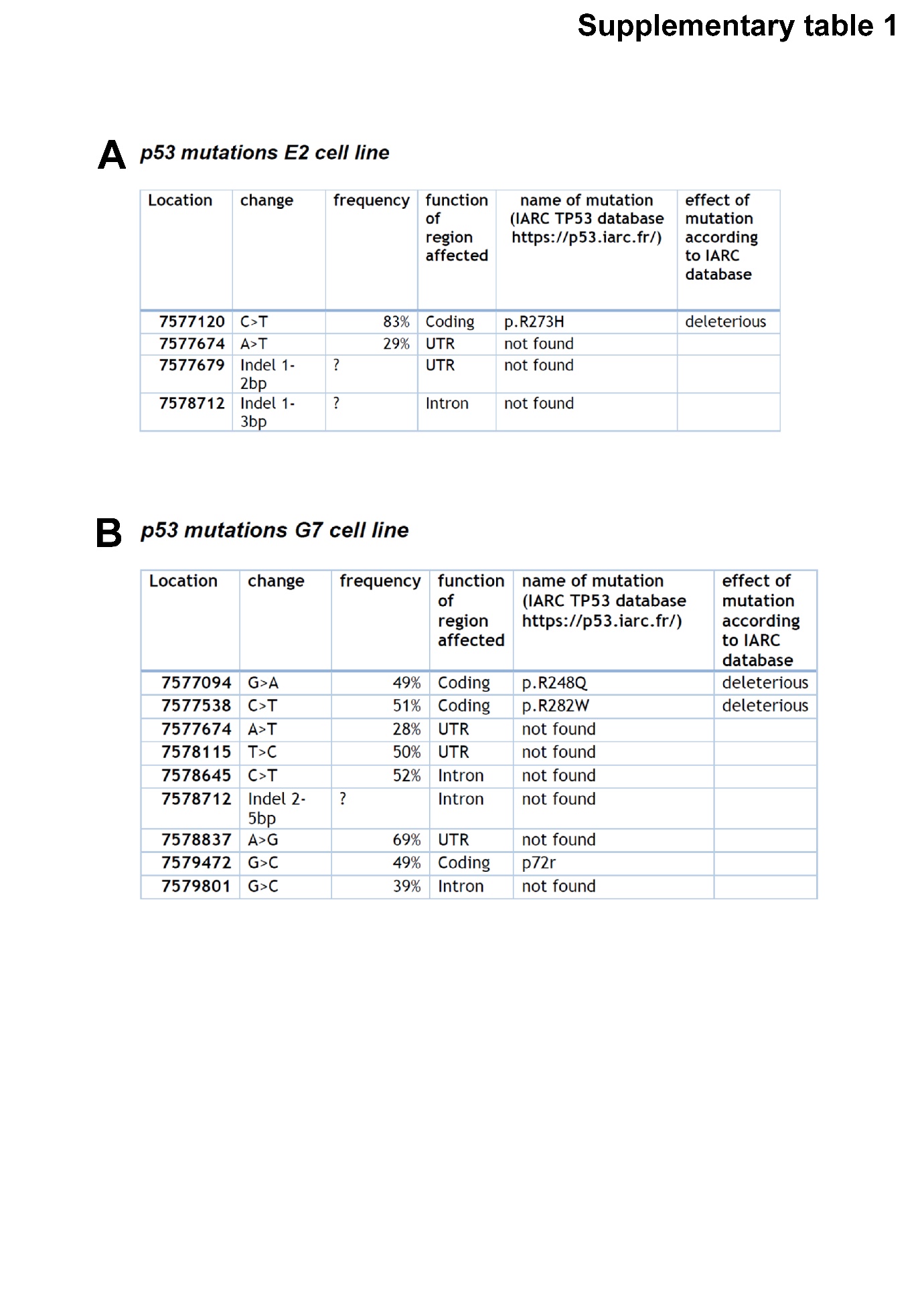
**

**Figure S1:**

**
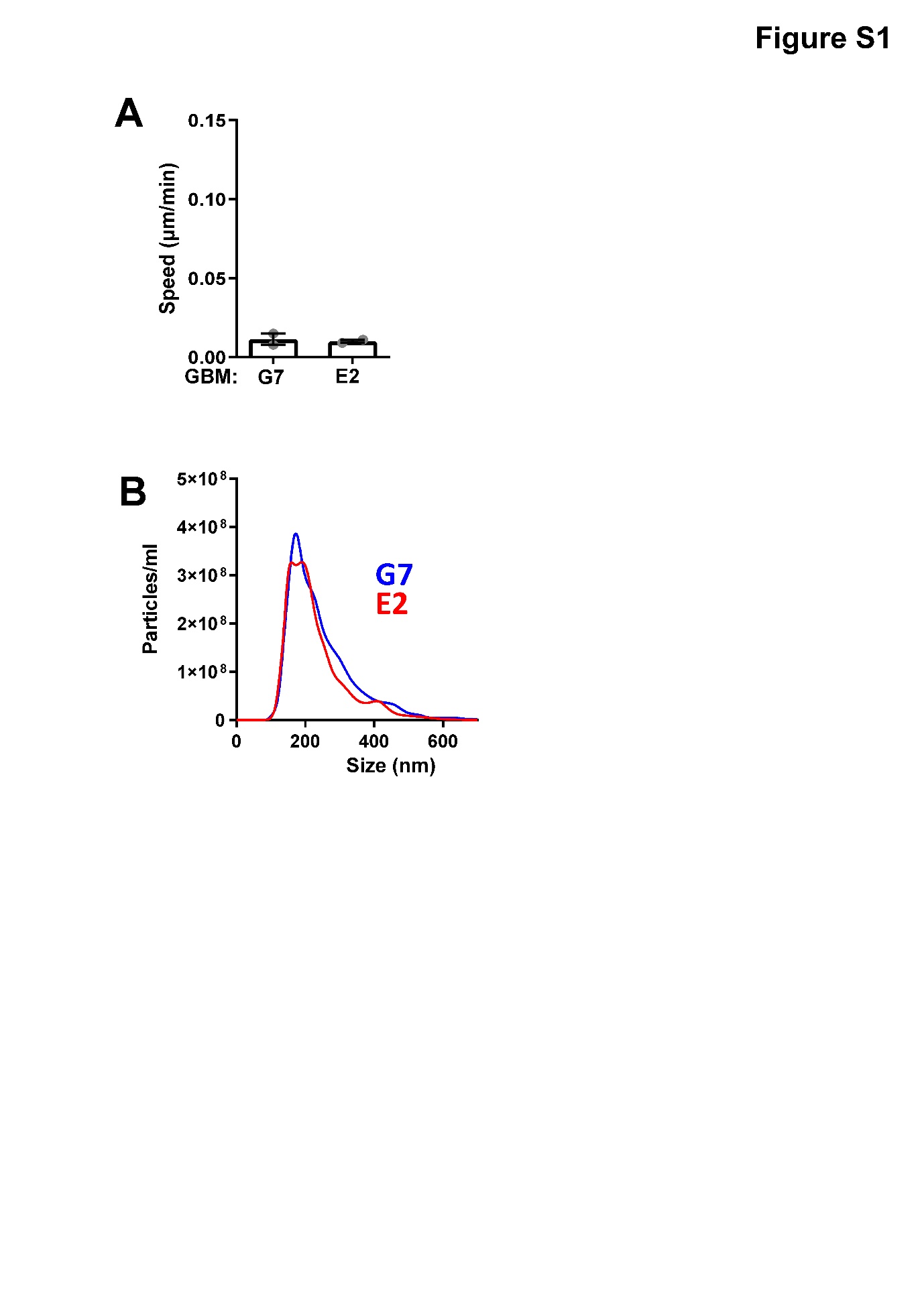
(A)**  **G7 and E2 GBM stem-like cells display similar migratory behaviour when plated onto mouse brain slices**

GFP-expressing GBM (E2 and G7) cells were plated onto coronal mouse brain slices and their migration speed determined using time-lapse fluorescence microscopy. Values are mean ± sem (n=2 independent experiments, 60 cell-tracks/condition/experiment).

**(B) Characterisation of EVs from G7 and E2 GBM cells**

E2 (red) or G7 (blue) GBM cells were incubated in EV-free medium for 48 hr. EVs were then purified from conditioned medium using differential centrifugation. The number and size-distribution of EVs was analysed using nanoparticle tracking. Each line represents the mean of three independent experiments.

**
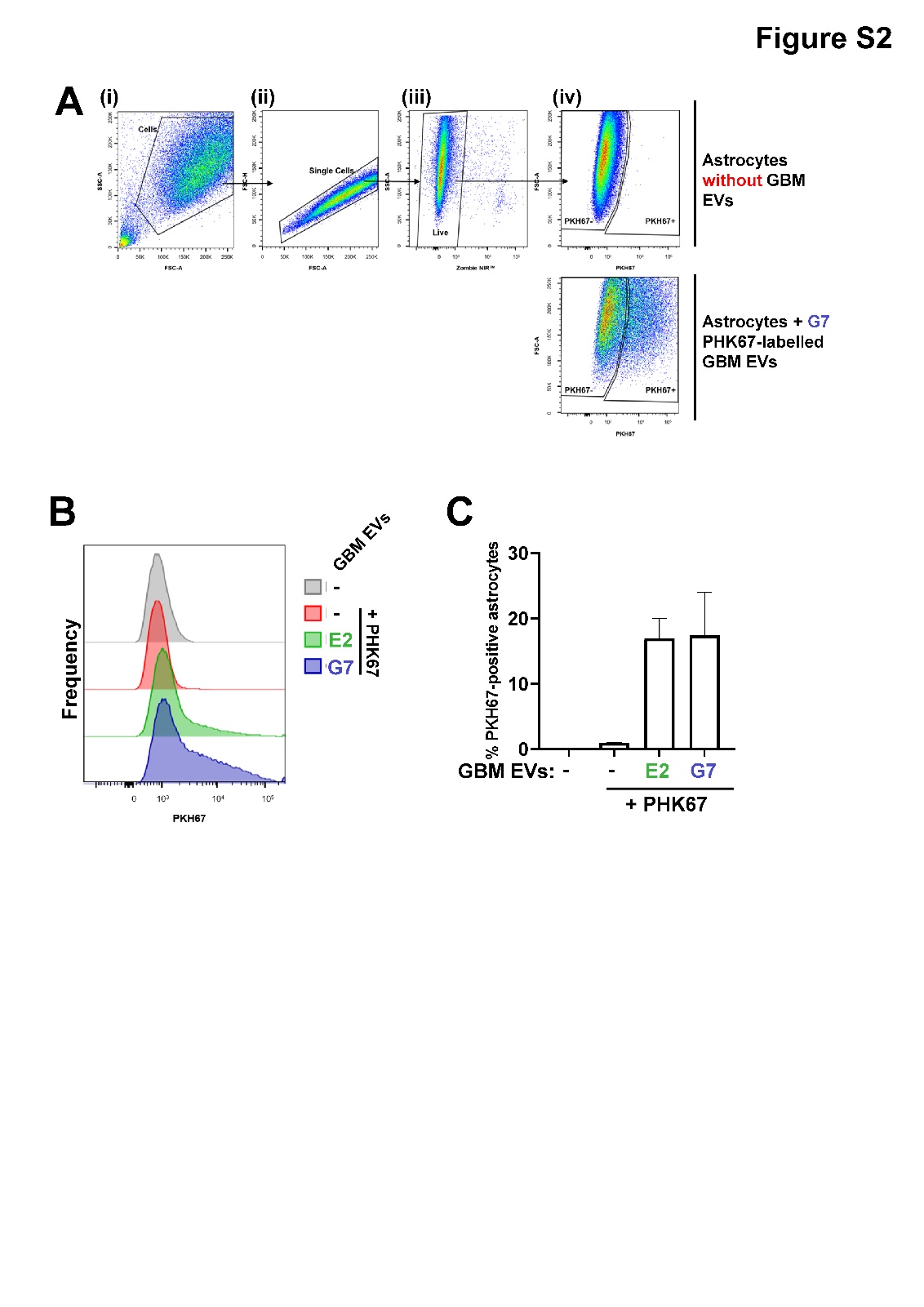
Figure S2:**

**Uptake of labelled EVs from G7 and E2 cells by astrocytes**

E2 (green) or G7 (blue) GBM cells were incubated in EV-free medium for 48 hr. EVs were then purified from conditioned medium using differential centrifugation, and then labelled by incubation with PHK67 (2μM) for 5 min. Excess dye was removed by ultracentrifugation (100,000 g for 70 min) and labelled EVs were added to primary cultured astrocytes for 24hr. Recipient astrocytes were then analysed using flow cytometry to determine uptake of labelled EVs. Flow cytometry was sequentially gated to include cells (**A**; (i)), single cells (**A**; (ii)) and live cells (**A**; (iii)), then PHK67 fluorescence was detected using an emission filter with 500 – 560 nm cut off (**A**; (iv)). The distribution of fluorescence intensities **(B)** and the proportion of recipient astrocytes which had taken-up PHK67-labelled EVs **(C)** from E2 (green) or G7 (blue) GBM cells was then determined. Values are mean ± SEM, n=6.

**
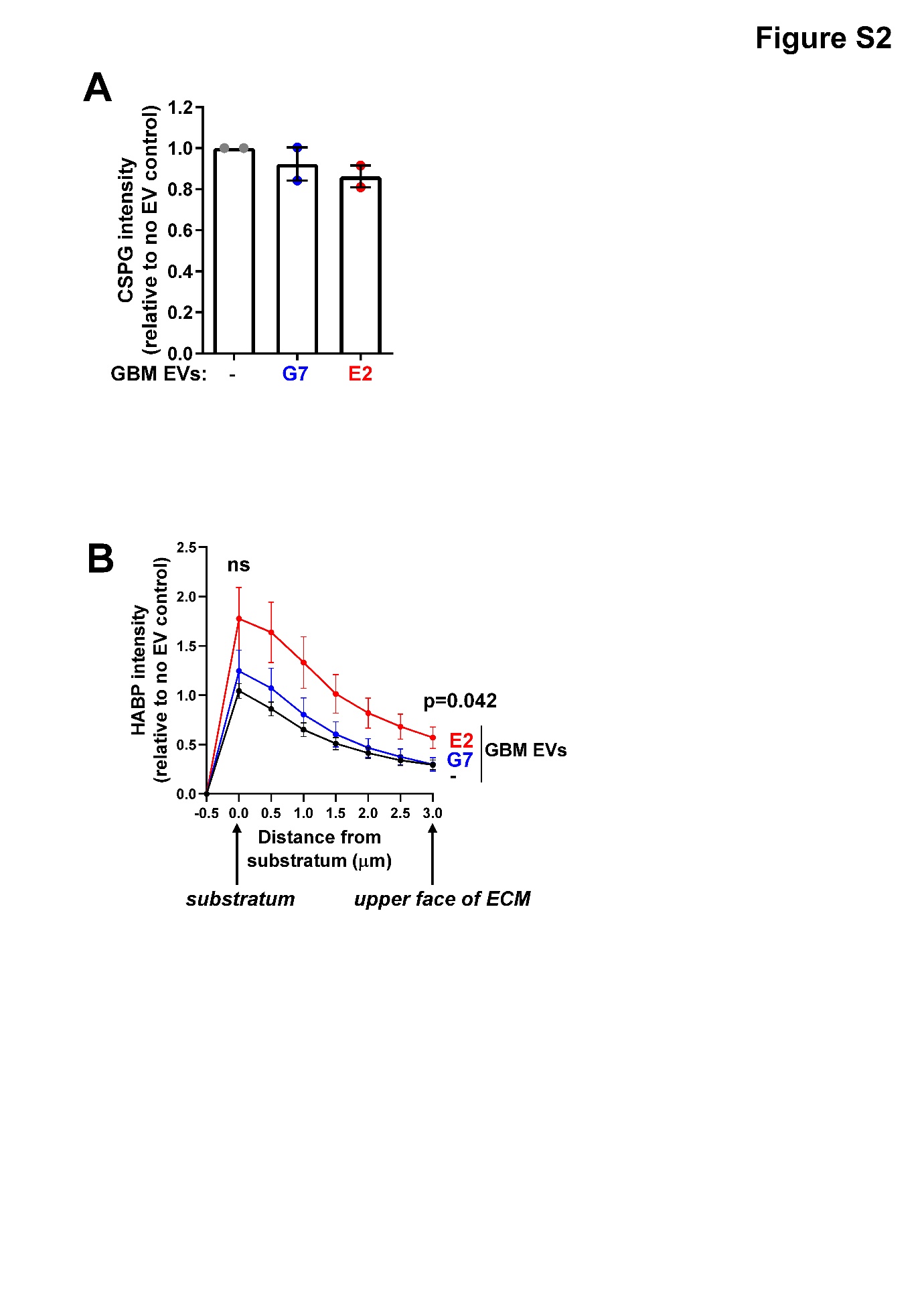
Figure S3:**

**(A) EVs from highly infiltrative E2 cells do not influence the CSPG content of brain slices**

Astrocytes were incubated with EVs from G7 or E2 GBM cells. Astrocytes were then allowed to deposit ECM for 6 days, stained with an antibody recognising CSPG and imaged using fluorescence confocal microscopy. Fluorescence was quantified using Image J. Values are mean ± sem (n=2 independent experiments, paired t-test; 7 fields/condition/experiment).

**(B) EVs from highly infiltrative E2 cells increase the HA content at the upper face of astrocyte-deposited ECM**

Astrocytes-deposited ECM was generated as for (A) and stained with biotinylated HABP followed by fluorescent streptavidin to visualize HA. The ECM was imaged using fluorescence confocal microscopy and the quantity of HA present in optical slices at the indicated distances from the substratum was determined using Image J. The upper face of the ECM is 3.0 μm from the substratum. Values are mean ± sem of three independent experiments, unpaired t-test E2 versus G7.

**Figure S4: Knockout of mp53^273H^ or Rab35 opposes growth of GBM in vivo**


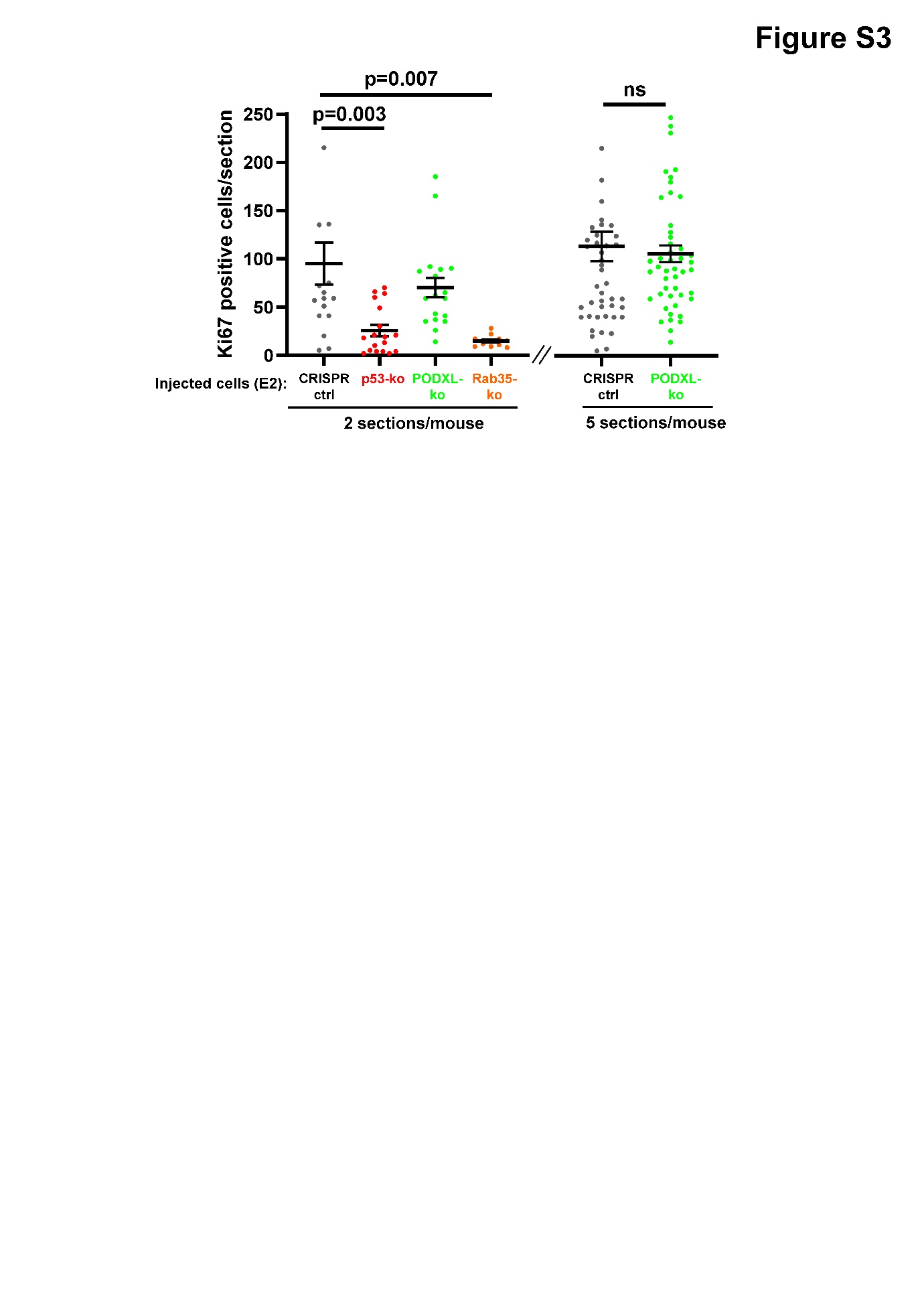
CRISPR control (CRISPR-ctrl) GBM (E2) cells, or those in which mp53^273H^ (p53-ko), PODXL (PODXL-ko) or Rab35 (Rab35-ko) had been knocked out were injected into the right striatum of CD1 nude mice. 9 weeks following injection, brains were fixed, cut into 50 μm sections and the quantity of tumour cells present in the section determined by staining for Ki67 followed by automated image analysis. The left- and right-hand graph represent data from 2 and 5 consecutive, 50 μm sections per mouse. Bars are mean ± sem. The dots represent the number of Ki67-positive cells present in each individual brain slice. Statistic is unpaired t-test with Welch’s correction, ns is not significant.

**
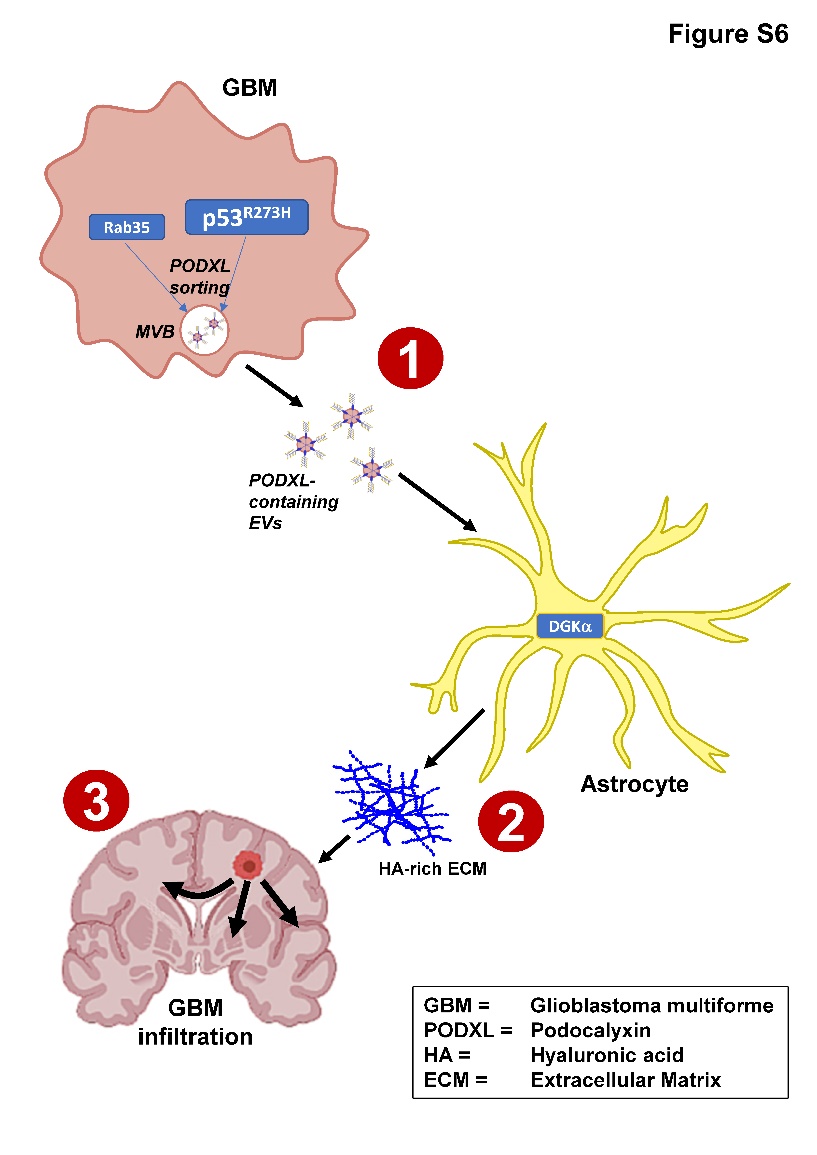
Figure S5: Schematic summary**

**[1]** In glioblastoma cells, a gain-of-function p53 mutation acts in combination with a GTPase of the Rab family (Rab35) to control sorting of a sialomycin, called podocalyxin, into small extracellular vesicles (EVs).

**[2]** Podocalyxin containing EVs from glioblastoma cells act on astrocytes to influence the type of extracellular matrix that they produce. They encourage astrocytes to deposit an extracellular matrix which is particularly rich in the glycan, hyaluronic acid (HA).

**[3]** HA-rich extracellular matrix, in turn, encourages the glioblastoma cells to be more invasive and to migrate long distances to infiltrate the brain.


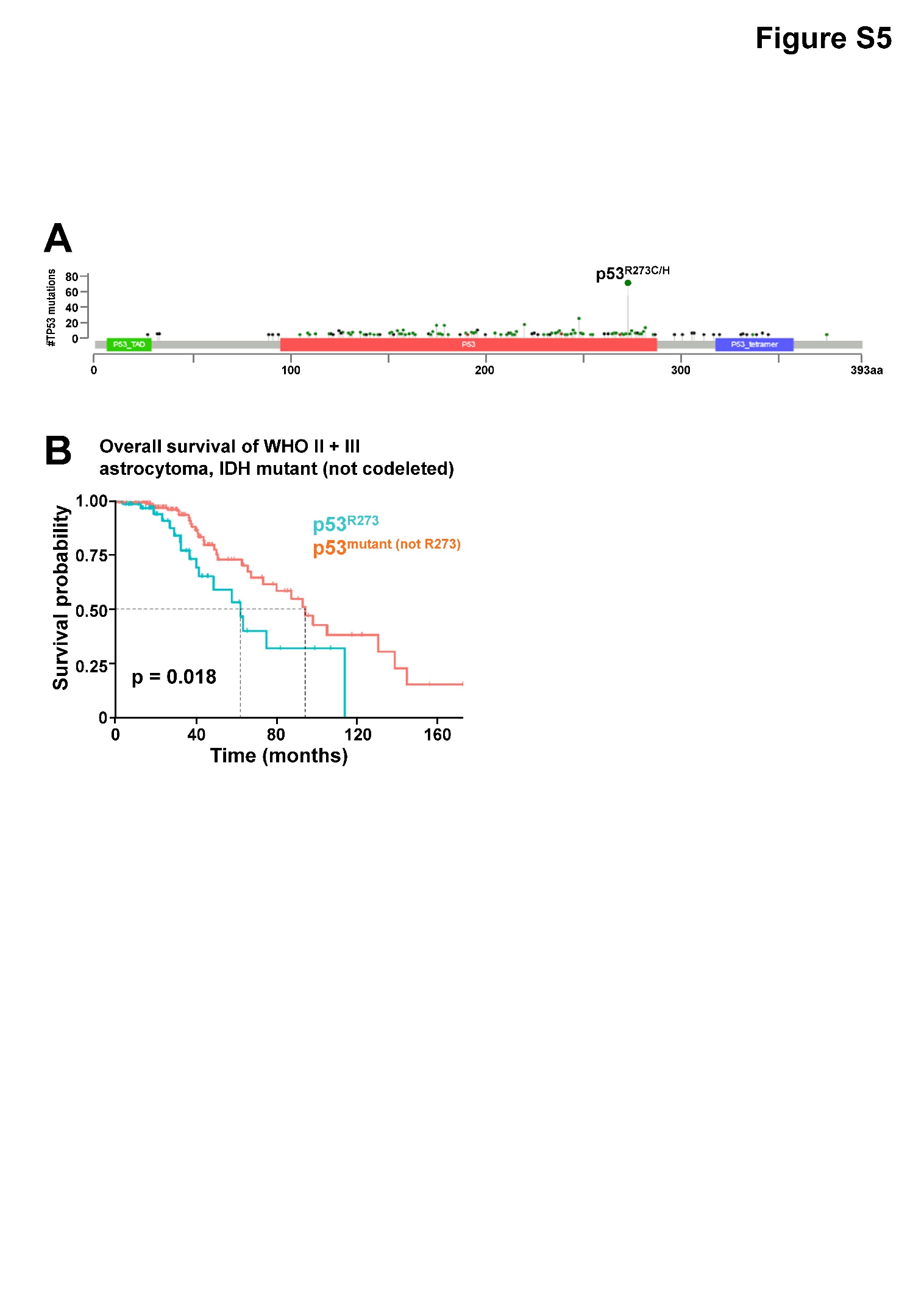
**Figure S6: p53 mutations in GBM**

**(A)** Distribution of p53 mutations within a merged TCGA cohort of LGG and GBM. The R273 locus, is by far the most common mutation across this panel of different tumours.

**(B)** Comparison of the overall survival of patients bearing p53^273H^ -expressing WHO II + III IDH mutant (not codeleted) astrocytoma (blue line) with patients bearing tumours of the same subtype, but that contained p53 mutations other than p53^R273H^ (orange line).
